# Dosimetric Superiority of High-Dose-Rate (HDR) Brachytherapy Using a Surface Mold Applicator for Primary Cutaneous Angiosarcoma of the Scalp

**DOI:** 10.7759/cureus.25056

**Published:** 2022-05-16

**Authors:** Kei Takase, Tomohiro Itonaga, Ryuji Mikami, Ryokichi Irisawa, Kazuhiro Saito

**Affiliations:** 1 Department of Radiology, Tokyo Medical University, Tokyo, JPN; 2 Department of Radiology, Tokyo Medical University Hospital, Tokyo, JPN; 3 Department of Dermatology, Tokyo Medical University, Tokyo, JPN

**Keywords:** high-dose-rate (hdr) brachytherapy, scalp tumors, vmat, radiotherapy (rt), angiosarcoma

## Abstract

The aim of this study was to demonstrate the utility of the dose-volume parameters of the target lesions and the radiation dose to the organ at risk (OAR) in two patients with primary cutaneous angiosarcoma of the scalp (CAS) treated with high-dose-rate brachytherapy (HDR-BT) using a surface mold applicator. In 2020, two men, aged 80 years and 60 years, respectively, were treated with HDR-BT with paclitaxel for CAS with no distant metastases and no surgical indication at our institution. The total irradiated dose was 57 Gy administered in daily fractions of 3 Gy four days per week. The method of HDR-BT involved the construction of a helmet that fitted over the patient's head, with parallel catheters fixed to the outside at 2.0 cm intervals to transport the iridium-192 HDR-BT source. In conclusion, HDR-BT may be superior when the dose to the target lesion plays a more important role than the OAR dose in selecting radiotherapy modalities for CAS.

## Introduction

Angiosarcomas is a rare soft tissue sarcoma of lymphatic or vascular endothelial cells origin. It is characterized by more than half of the cases occurring in the head and neck region [1,2]. Risk factors for angiosarcoma have been reported to include ultraviolet light, radiation therapy, race, and advanced age [3,4]. Primary cutaneous angiosarcoma of the scalp (CAS) typically presents as purpura, often multiple, and sometimes forms bleeding and ulcers. In the treatment of patients with locally advanced CAS, surgery is the first choice but is more likely to result in positive margins due to a strong invasion of the surrounding tissue [5]. Therefore, multidisciplinary treatments such as surgery, radiotherapy, and chemotherapy are used, but even in recent years, the prognosis is poor, with a five-year survival rate of 33.6-43% [4,6,7].

In the treatment of CAS, radiation therapy is used both as curative and adjuvant, but there is controversy about optimal irradiation methods and prescribed doses. Conventional external beam radiotherapy (EBRT) has been used for CAS in previous studies, but with the complex shape of the scalp, there was a drawback that the dose to the target and the organ at risk (OAR) were trade-offs [8,9]. We used high-dose-rate brachytherapy (HDR-BT) with a surface mold applicator to solve this problem and reported good results [10]. On the other hand, there has been an increasing number of reports in recent years on the use of intensity-modulated radiation therapy (IMRT) to solve this problem even with EBRT. Clinical studies have reported improved uniformity of dose distribution, especially for volumetric-modulated arc therapy (VMAT), while reducing the dose to the OAR compared with coplanar/non-coplanar IMRT [11]. In this report, we present two cases where we used HDR-BT against CAS, and we then compared the dose-volume parameter for the target lesion and the irradiation dose to the OAR with previous reports in the literature.

## Case presentation

Case 1

**Figure 1:**
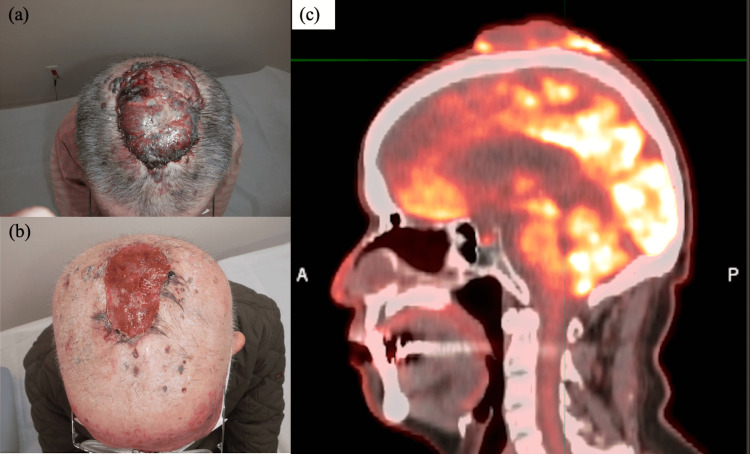
Case 1: (a) Scalp at initial examination; (b) Scalp during treatment (51Gy/17Fr); (c) Pre-treatment PET-CT. PET-CT: positron emission tomography-computed tomography

An 80-year-old man presented with the chief complaint of a red skin rash with bleeding on the parietal region that had been increasing for about four months. The patient was referred to our center because angiosarcoma was suspected on visual examination. On initial examination, a 120 mm x 60 mm erythematous plaque was observed on the parietal region, and a black nodule with an 80 mm x 50 mm ulcer was observed in the center (Figure 1). A biopsy of this region was performed, and histological examination revealed spindle-shaped cells proliferating in a chordate to the reticular pattern between existing collagen fibers. Microscopic hemorrhage foci were observed. Immunostaining showed CD31 (+), CD34 (±), D2-40 (+), S100 (-), and Ki-67 labeling index 50%, and the diagnosis of angiosarcoma was made. The patient underwent 18F-fluorodeoxyglucose-positron emission tomography for staging purposes. Consistent with the findings on visual examination, there was an accumulation of a maximum standardized uptake value (SUVmax) of 12.68 in the parietal region. No obvious distant metastasis could be noted. According to the American Joint Committee on Cancer (AJCC) tumor, nodes, and metastases (TNM) staging, the patient was diagnosed as T3N0M0 Stage III. There were no adverse events other than radiation dermatitis consistent with the irradiated field, and the 57 Gy/19 Fr treatment was completed as planned (Figure 1).

Case 2

**Figure 2:**
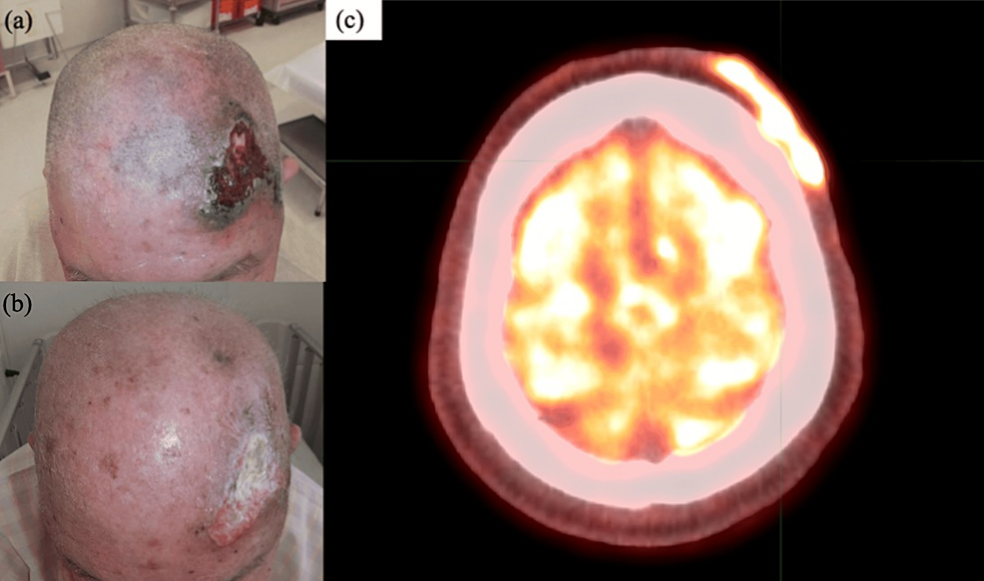
Case 2: (a) Scalp at initial examination; (b) Scalp during treatment (45Gy/15Fr); (c) Pre-treatment PET-CT. PET-CT: positron emission tomography-computed tomography

A 69-year-old man presented in 2020 with the chief complaint of a red skin rash with bleeding on the left frontal region that had been increasing for about five months. The patient was referred to our center because angiosarcoma was suspected on visual examination. On initial examination, a 60 mm x 60 mm purpura with a central ulcer on the left frontal region and a thickened purpura in the parietal region was observed (Figure 2). A biopsy of the area was performed, and histological examination showed spindle-shaped tumor cells proliferating from the dermis in all layers, with findings of red blood cell leakage in the stroma. Microscopic hemorrhage foci were observed. Immunostaining showed CD31 (+), CD34 (-), D2-40 (+), Factor VIII (+), and Ki-67 labeling index 40%, and the diagnosis of angiosarcoma was made. The patient underwent 18F-fluorodeoxyglucose-positron emission tomography for staging purposes. Consistent with the findings on visual examination, there was an accumulation of SUVmax 11.99 in the left frontal region. No obvious distant metastasis could be noted. According to the AJCC TNM staging, the patient was diagnosed as T2N0M0 Stage II. There were no adverse events other than radiation dermatitis consistent with the irradiated field, and the 57 Gy/19 Fr treatment was completed as planned (Figure 2).

Brachytherapy treatment

**Figure 3:**
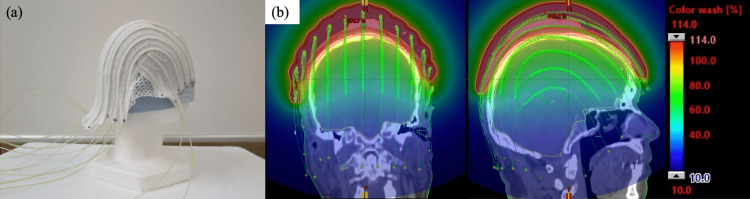
(a) HDR-BT with a surface mold applicator; (b) Location of radiation sources and dose distribution in the RTPS for Case 1 HDR-BT: high-dose-rate brachytherapy; RTPS: radiation treatment planning system

We performed chemotherapy and HDR-BT in combination for the patients as they had extensive lesions with unclear borders, and were deemed inoperable. The chemotherapy regimen was paclitaxel weekly at a dose of 60 mg/m2 (given six weeks on/two weeks off). We used HDR-BT with the surface mold technique to prescribe homogenous doses for complex shapes of the scalp. The details of HDR-BT are described in a previous paper [10]. Iridium-192 HDR remote afterloading system (RALS) is applied to this technique (VariSource™ iX afterloader, Varian Medical Systems, Inc., Palo Alto, California, United States). First, it made helmet-type molds with heat-moldable plastic mesh according to the shape of the individual patients in a sitting position (Figure 3).

Next, polyethylene catheters were fixed parallel with the interval of 2.0 cm outside the helmet. In consultation with a dermatologist, we marked an irradiation field on the skin and mask. The mold applicator was attached to the patient’s head, and a CT scan was done. Treatment plans were created based on the CT images. Dose calculation of brachytherapy was done using BrachyVision (Varian Medical Systems, Palo Alto, California, United States). Active dwell positions of 5 mm length source were located at 3 mm intervals in each source into catheters to ensure adequate dose distribution and clinical target volume coverage. The placements of reference points were set on the surface of the scalp. Lenses, brain, and skin were defined as OAR. The target volume setting was determined by consensus between the dermatologist and radiation oncologist. The area in which tumors were evident on visual examination and imaging was defined as gross tumor volume (GTV), and areas surrounding the GTV that were at risk of invasion were defined as clinical target volume (CTV). Brachytherapy for CAS does not need to consider inter/intra fractional organ motion or setup error, and CTV and planning target volume (PTV) were identical. We designed the treatment plan to cover 95% of the target lesion with 95% of the prescribed dose for PTV and 100% of the target lesion with 90% of the prescribed dose for GTV. The total irradiated dose was 57 Gy administered in daily fractions of 3 Gy four days per week. Doses for OAR were set so that the maximum point dose (Dmax) of the cerebrum would not exceed the prescribed dose (57 Gy) and the Dmax of the eyeball would not exceed 35 Gy. 

Dose evaluation

The doses received by 98%, 95%, 90%, 50%, 10%, and 2% of the volume in PTV and GTV (D98, D95, D90, D50, D10, D2) were measured. Similarly, the volumes of target lesions receiving 150%, 100%, and 90% of the prescribed dose (TV150, TV100, and TV90) were also measured. Regarding the irradiation dose of OAR, Dmax of the eyeball and Dmax, mean dose, and D2cc of the cerebrum were examined.

The following indices were used to measure the conformity index and homogeneity index to the target volume in treatment planning. We adopted the conformal index (COIN), which considers the dose to OAR in addition to the conformation number devised by Baltas et al. [12]. 

$COIN=\frac{TV_{ref}}{TV}\times \frac{TV_{ref}}{V_{ref}}\times \prod_{i=1}^{N_{co}}\left [ 1-\frac{V_{coref,i}}{V_{co,i}} \right ]$ 

Where TV is the target volume, TV_Ref_ is the target volume covered by the prescribed dose, V_Ref_ is the volume of the prescribed dose, V_CO,i_ is the volume of the OAR, V_COref,i_ is the volume of the OAR receiving at least the prescribed dose, and N_co_ is the number of OAR. As a homogeneity index, brachytherapy adopted the dose homogeneity index (DHI) [13]. 

$DHI=\frac{\left ( TV_{ref} -TV_{150}\right )}{TV_{ref}}$

The closer the value of DHI is to one, the higher the homogeneity is. The brachytherapy has the dose non-uniformity ratio (DNR), which is an index of dose uniformity used in the interstitial method [14]. DNR is sometimes used as a complementary parameter to DHI (DNR=1-DHI) with the lower value of DNR indicating a more homogeneous dose distribution.

Result

There were no findings suggestive of recurrence during the second half-year follow-up of radiotherapy in either case. The dosimetric parameters to target lesions and OAR for HDR-BT are shown in Table 1.

**Table 1:** Comparison of dosimetric parameters between target lesions and OARs in cases PTV: planning target volume; VPTV: volume of PTV; GTV: gross tumor volume; Dmax: maximum point dose; OAR: organs at risk

	Case 1	Case 2
PTV volume	287.7 cc	336.0 cc
PTV min	69.3 %	68.0 %
PTV D_98_	89.4 %	90.0 %
PTV D_95_	95.1 %	96.3 %
PTV D_90_	99.1 %	98.9 %
PTV D_50_	109.6 %	108.2 %
PTV D_10_	129.6 %	122.7 %
PTV D_2_	150.8 %	138.1 %
PTV mean dose	112.4 %	109.8 %
PTV_90_ (VPTV_90_)	281.3 cc	329.3 cc
PTV_100_(VPTV_100_)	252.7 cc	290.9 cc
PTV_150_(VPTV_150_)	6.1 cc	2.3 cc
GTV volume	122.9 cc	19.3 cc
GTV min	91.7 %	97.6 %
GTV D_98_	97.4%	107.7 %
GTV D_95_	98.9 %	109.4 %
GTV D_90_	100.4 %	111.2 %
GTV D_50_	109.6 %	119.3 %
GTV D_10_	129.8 %	133.9 %
GTV D_2_	146.2 %	149.6%
GTV mean dose	112.7 %	121.3 %
GTV_90_ (VGTV_90_)	122.9 cc	19.3 cc
GTV_100_ (VGTV_100_)	112.4 cc	19.3 cc
GTV_150_ (VGTV_150_)	1.7 cc	0.4 cc
Cerebrum D_max_	54.4 Gy	51.5 Gy
Cerebrum D_2cc_	51.0 Gy	49.5 Gy
Cerebrum mean dose	29.4 Gy	34.0 Gy
Eye D_max_	30.2 Gy	31.7 Gy

There was no volume of OAR irradiated above the prescribed dose. The COIN of PTV was 0.68 in Case 1 and 0.60 in Case 2. The DHI of PTV was 0.992 and 0.976 in Case 1 and Case 2, respectively.

## Discussion

In this study, we investigated the dosimetric superiority of HDR-BT with a surface mold applicator for CAS using two cases. At our institution, we perform HDR-BT for CAS with daily fractions of 3 Gy three days per week for a total dose of 60 Gy. The coronavirus disease 2019 (COVID-19) epidemic, however, called for a reduction in the duration of radiotherapy, so instead of reducing the total dose, the number of treatments per week was increased by one day. The patients' progress in the first six months after treatment has been good, with no adverse events exceeding grade 3 and no evidence of recurrence.

There are several previous studies that have performed HDR-BT with surface mold technique against CAS like the present study [15,16]. As compared with the conventional EBRT, the surface mold technique could be used to treat irregular or curved surfaces like the head, which makes it possible to uniform dose prescription to the tumor. Our facility applied the report of Imai et al. [15]. In contrast to the original applicator, our method has changed the placement of the catheter to sagittal from the coronal direction because of the advantage of being able to prescribe a homogeneous dose for occipital tumors. In addition, the distance between the catheters was set at 2 cm. The reason for this spacing is to avoid producing an extremely high dose region to the normal tissue between the catheters. The dosimetric advantage of using the surface mold applicator method against CAS is that V150 is lower than that of the interstitial method. In breast brachytherapy, a significant relationship between dose uniformity and the cosmetic outcome has been reported, with higher values of DHI associated with less late fibrosis [17]. This approach may lead to a reduction in late adverse events, as the DHI was above 0.9 in both of our cases.

A comparison of HDR-BT and EBRT for skin tumors of the head and neck reported that HDR-BT was superior in COIN, indicating dose conformity [18]. It is known that the rate of local control of angiosarcoma correlates with a higher dose and, for the same dose prescription, brachytherapy may be an excellent option for sarcomas with relatively low alpha/beta ratios [1,19]. The OAR doses were then compared by HDR-BT and VMAT methods. The only previous study comparing HDR-BT and VMAT OAR doses to CAS showed that VMAT had a significantly lower brain dose (Dmax, D2cc) and eye Dmax [20]. The OAR doses of our study are all higher than in this previous literature, indicating that VMAT may be useful for dose reduction to OAR.

## Conclusions

In this study, we analyzed the superiority of HDR-BT on dose-volume parameters analysis for two cases of CAS. Although the results were based on a small number of patients, HDR-BT may be superior when the dose to the target lesion plays a more important role than OAR dose in selecting radiotherapy modalities for CAS. Future comparisons should be made using a larger number of cases.
